# Diaminoanthraquinone Enhances Alkaloid Ionization in MALDI-MS

**DOI:** 10.5702/massspectrometry.A0109

**Published:** 2022-12-28

**Authors:** Tohru Yamagaki, Tsukiho Osawa

**Affiliations:** 1 Bioorganic Research Institute, Suntory Foundation for Life Sciences, 8–1–1 Seikadai, Seika-cho, Soraku-gun, Kyoto 619–0284, Japan

**Keywords:** ionization mechanism, MALDI, alkaloids

## Abstract

The alkaloids epinastine, 3-methylxanthine and camptothecin were analyzed by matrix-assisted laser desorption/ionization mass spectrometry (MALDI-MS). The ionization efficiencies of epinastine and 3-methylxanthine were improved upon the addition of 1,5-diaminoanthraquinone (DAAQ). DAAQ did not show ultraviolet absorbance peaks at wavelengths around 337 nm and 355 nm that are used in conventional MALDI-MS instruments. In addition, the DAAQ ion peak was very weak relative to those of the analytes due to the low absorbance efficiency. These properties of DAAQ are advantageous for the DAAQ-MALDI-MS analysis of alkaloids.

## INTRODUCTION

Matrix-assisted laser desorption/ionization (MALDI) is a method for ionizing non-volatile biological samples and involves mixing a sample with a small organic molecule matrix that absorbs laser light. MALDI mass spectrometry (MS) is an essential technique in the life sciences because it permits a wide range of biomolecule analyses such as proteins, peptides, metabolites, and glycans. The chemical properties of the matrix are important for the analysis of biomolecules using MALDI-MS, and optimal matrices have been developed for each type of biomolecule through the combination of past experiences including trial-and-error experiments. α-Cyano-4-hydroxy-cinnamic acid (CHCA) is widely used for peptide analysis,^1)^ dihydroxybenzoic acid (DHB) is used for glycan analysis^2)^ and 3,5-dimethoxy-4-hydroxycinnamic acid (SA) is used for the analysis of large proteins.^3)^

The details of the ionization mechanism in MALDI are generally considered to be as follows.^4–7)^ When the UV-absorbed matrix is excited by laser irradiation, it is rapidly heated and explosively desorbed and vaporized. At the same time, the biological sample which is dispersed in the large amount of matrix is vaporized. Electrons and protons are then transferred in the vaporized matrix and the ionized analytes form a plume-like molecular cloud. The ionized molecules are extracted by an applied voltage and separated based on their masses in a vacuum flight tube followed by detection. It is generally assumed that matrices should be small organic molecules that absorb at the wavelength of the commonly used nitrogen laser (around 337 and 355 nm) and should have a low boiling point. To this end, chemical compounds with the UV absorption characteristics described above have been investigated for use as matrices.^8)^

We previously reported on the analysis of alkaloids by MALDI-MS.^9)^ Some alkaloids can be ionized in matrix-free MALDI, that is, laser desorption/ionization (LDI) MS. This is probably due to their UV absorption properties and the presence of nitrogen as a heteroatom. In LDI the efficiency of ionization is dependent on the alkaloid under consideration; some alkaloids are ionized easily even with weak laser excitation, while others are ionized only with strong laser excitation or are extremely difficult to ionize. Because of this, we have been searching for new types of ionization-assisting compounds as a new chemical category for the analysis of alkaloids by MALDI-MS.

Our focus was on the laser fluence threshold for alkaloids with or without a matrix because the relationship between the ion signals and the laser fluence is an important factor in MALDI.^10)^ Laser fluence in MALDI is the laser strength per square meter needed to ionize the sample and the matrix. Laser fluence is directly related to the laser power of the available instruments because the laser diameter is fixed in this study.

1,5-Diamino anthraquinone (DAAQ) is a red dye and an industrially important compound. DAAQ has an absorption peak at a UV wavelength of around 500 nm and no absorption peak around 337 and 355 nm (Supporting Information-1; SI-1). There was no wavelength shift in the UV spectra for a mixture of DAAQ and an alkaloid (Supporting Information-2). Rhodamine dyes with 3-nitrobenzyl alcohol were previously used as matrix for the MALDI-MS analysis of proteins using a 532 nm wavelength laser system.^11)^ It was assumed that DAAQ had no function as a matrix in a conventional UV laser system (337 or 355 nm) in MALDI-MS. It should be noted, however, that DAAQ has been reported to induce the visible-light catalytic photooxygenation of alkaloids.^12)^ We found that DAAQ improved the ionization efficiency of alkaloids when mixed with alkaloids in MALDI-MS analyses.

## MATERIALS AND METHODS

### Materials

DAAQ (melting point, 319°C) and 3-methylxanthine (m.p. >300°C) were purchased from Tokyo Chemical Industry CO., LTD., Tokyo, Japan. Epinastine (m.p. 205–208°C) was purchased from BLD Pharmatech Ltd., Shanghai, P.R. China. Camptothecin (m.p. 275–277°C) was purchased from Alomone Labs, Israel. Methanol (HPLC grade) was purchased from NACALAI TESQUE, INC., Kyoto, Japan. Water was produced by an ultrapure water production system from Milli-Q IQ 7003 (Merck KGaA, Darmstadt, Germany) with ion-exchange filtration by activated carbon.

5 mM DAAQ was prepared in 60% methanol. 2 mM Epinastine, camptothecine, and 3-methylxanthine were prepared in 60% methanol.

The analytes were premixed with a 5 mM DAAQ solution or a 60% methanol solution (50 : 50, v/v), and a 1 μL aliquot of the premixed analyte solution was deposited on the MALDI-MS plate.

### Ultraviolet-visible absorption spectrometer

Ultraviolet-visible absorption (UV-Vis) spectra were acquired using a UV-Vis JASCO V-650 spectrophotometer (JASCO Corporation, Tokyo, Japan). A disposable plastic cuvette was used with a 1 cm optical path length. 50 μM DAAQ was prepared in 60% methanol for Supporting Information-1. 100 μM DAAQ and 40 μM epinastine were prepared in 60% methanol for Supporting Information-2.

### Mass spectrometry

All MS spectra were acquired on a RapifleX MALDI-TOF/TOF tandem MS/MS instrument (Bruker Corporation, Billerica, MA, USA) in the reflectron and positive-ion mode in the 0–2 kDa range. The laser was a Smartbeam 3D laser with a wavelength of 355 nm and a laser energy of >100 μJ/pulse. The ion acceleration was 20 kV as ion source 1, the lens was 11.5 kV, and reflectron 1 was 20.83 kV. Mass spectra of 10000 laser shots were accumulated from 2000 laser shots in different areas. The laser irradiation power was controlled with an attenuation filter, and the power values are shown as the percentage (%) of the laser output (100%).

## RESULTS AND DISCUSSIONS

**Figure 1:**
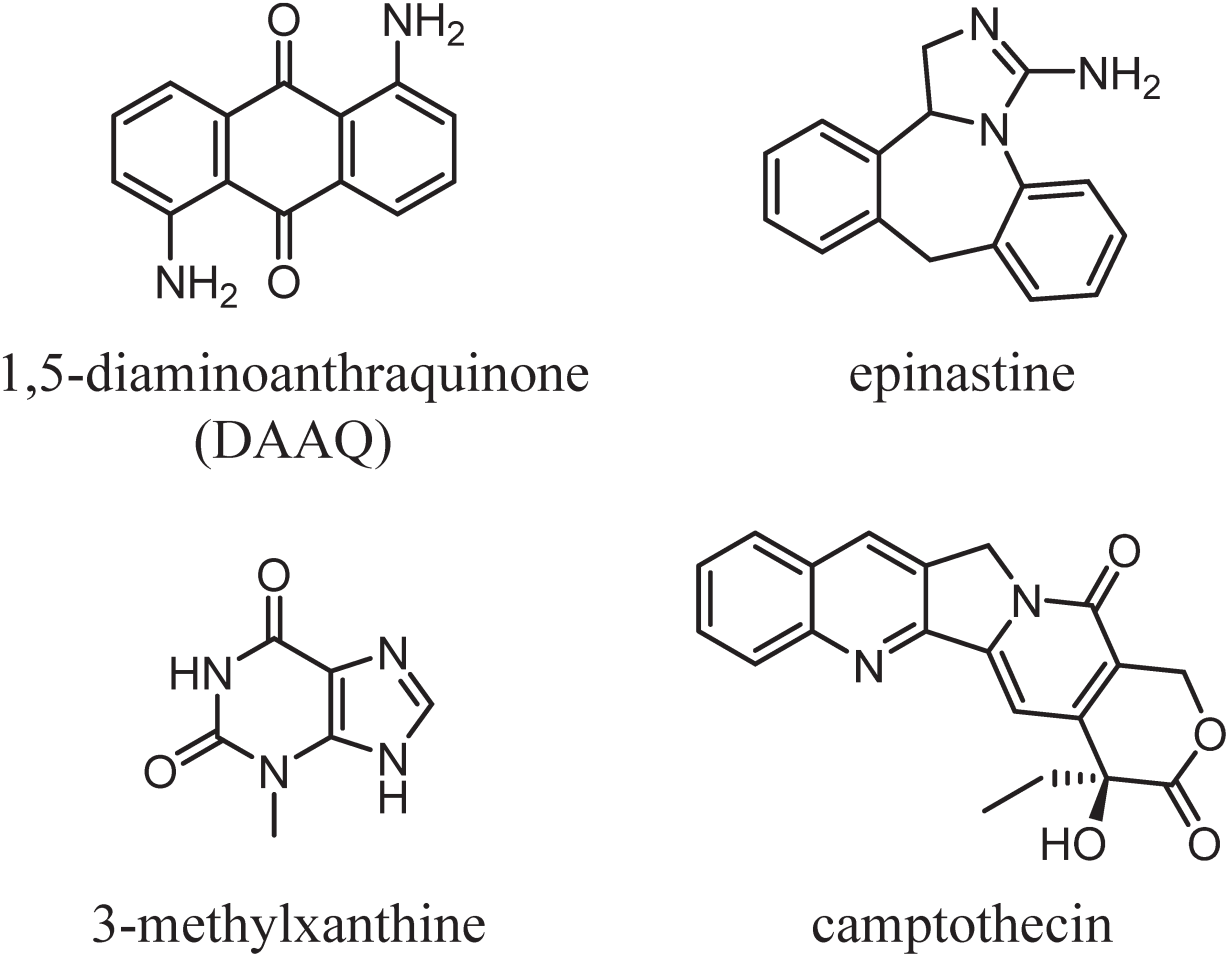
Fig. 1. Structure of diaminoanthraquinone, epinastine, 3-methylxanthine, and camptothecine.

Figure 1 shows the structures of DAAQ (C_14_H_10_N_2_O_2_; the mono isotopic molecular mass is 238.0742), and analyte alkaloids epinastine (C_16_H_15_N_3_; 249.1266), 3-methyl xanthine (C_6_H_6_N_4_O_2_; 166.0491) and camptothecine (C_20_H_16_N_2_O_4_; 348.1109). The ionization efficiency was estimated from the threshold of the laser irradiation power in the MALDI-MS of the analyte alkaloids because the more easily ionized compounds would be ionized by the lower laser fluence.

**Figure 2:**
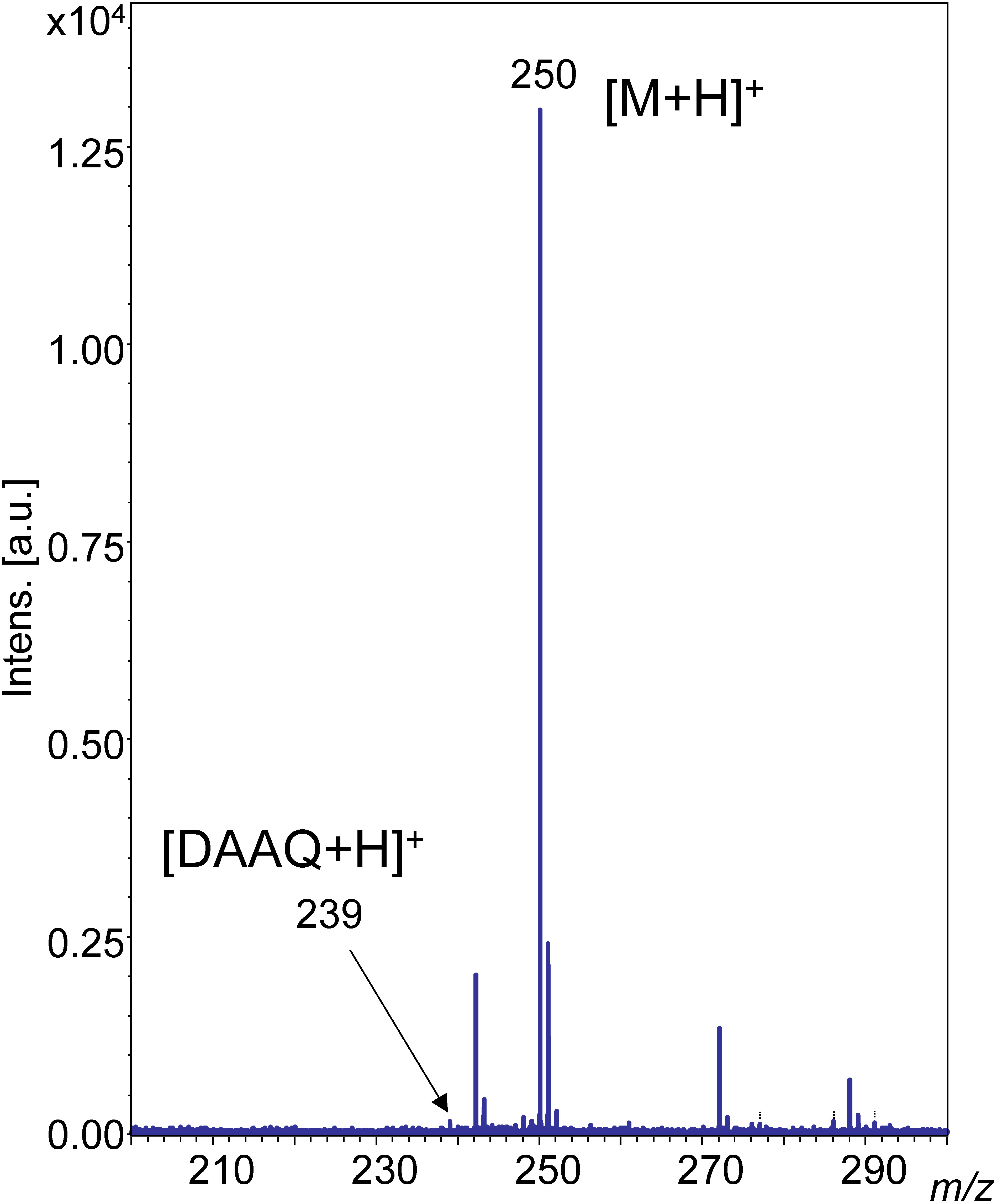
Fig. 2. DAAQ-MALDI mass spectrum of epinastine at 20% laser power.

**Figure 3:**
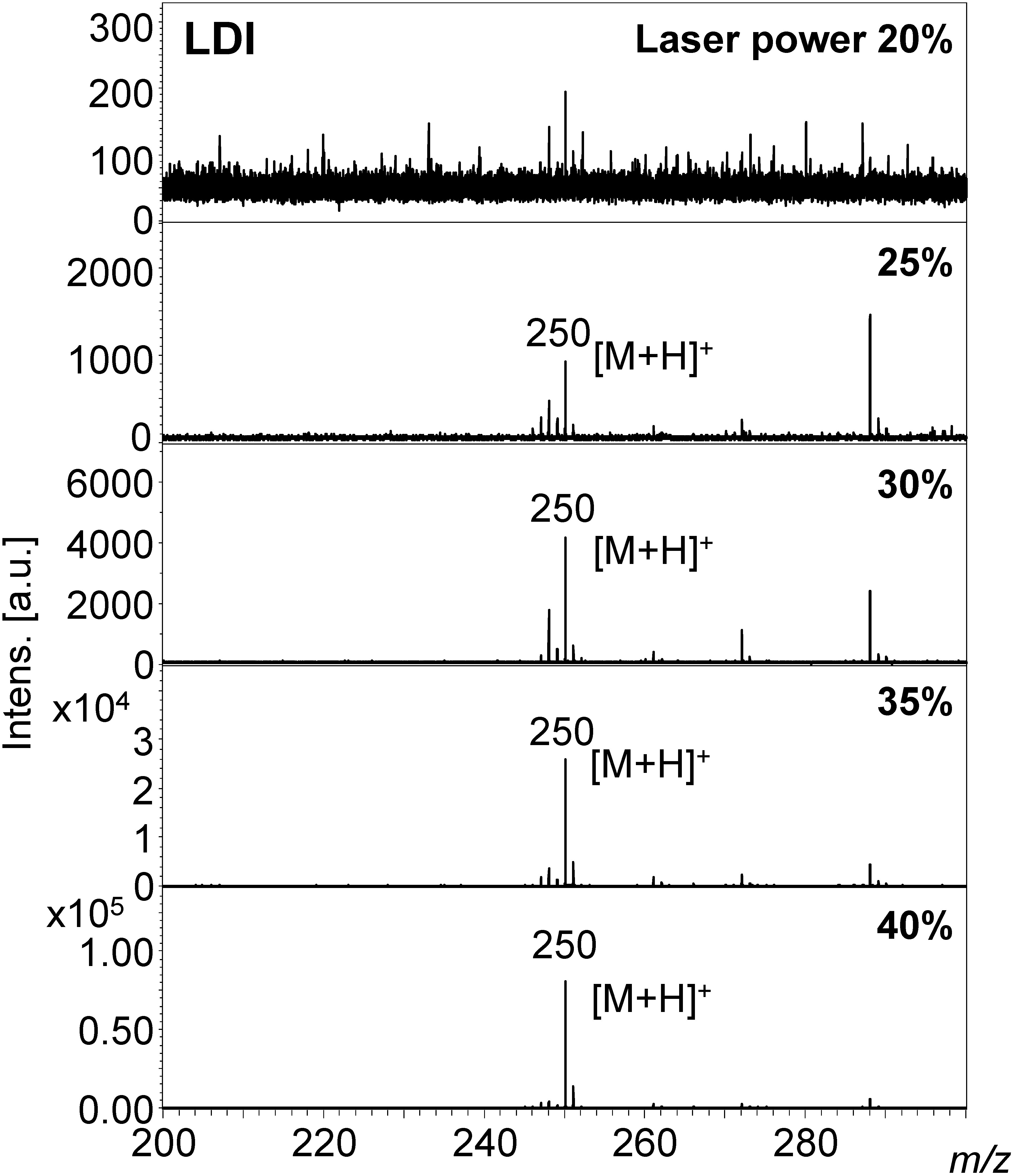
Fig. 3. LDI mass spectra of epinastine at 20%–40% laser power.

A protonated molecule [M+H]^+^ was observed in the MALDI-MS spectrum of epinastine with DAAQ at *m*/*z* 250. The signal to noise ratio (*S*/*N*) was high at a low laser power of 20%, as shown in Fig. 2. In contrast, the *S*/*N* value was low in the case of the LDI-MS of epinastine without DAAQ at the same laser power of 20%, as shown in Fig. 3. A laser power of 35–40% was needed to obtain the same quality mass spectrum in LDI-MS compared with the DAAQ-MALDI MS of epinastine. 100 pmol epinastine could be detected by MALDI-MS as the concentration of epinastine was reduced (Supporting Information-3).

**Figure 4:**
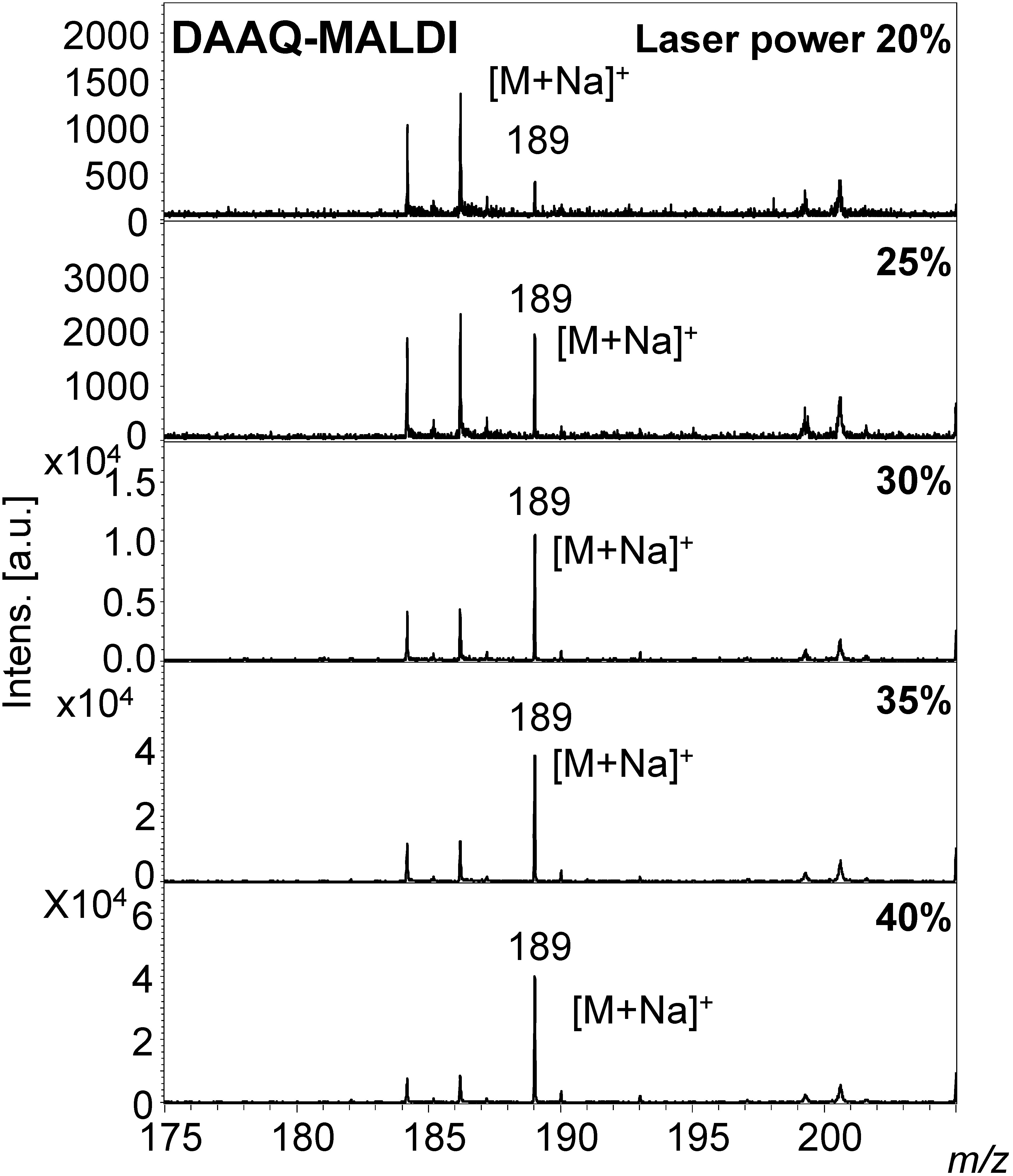
Fig. 4. DAAQ-MALDI mass spectra of 3-methylxanthine at 20%–40% laser power.

**Figure 5:**
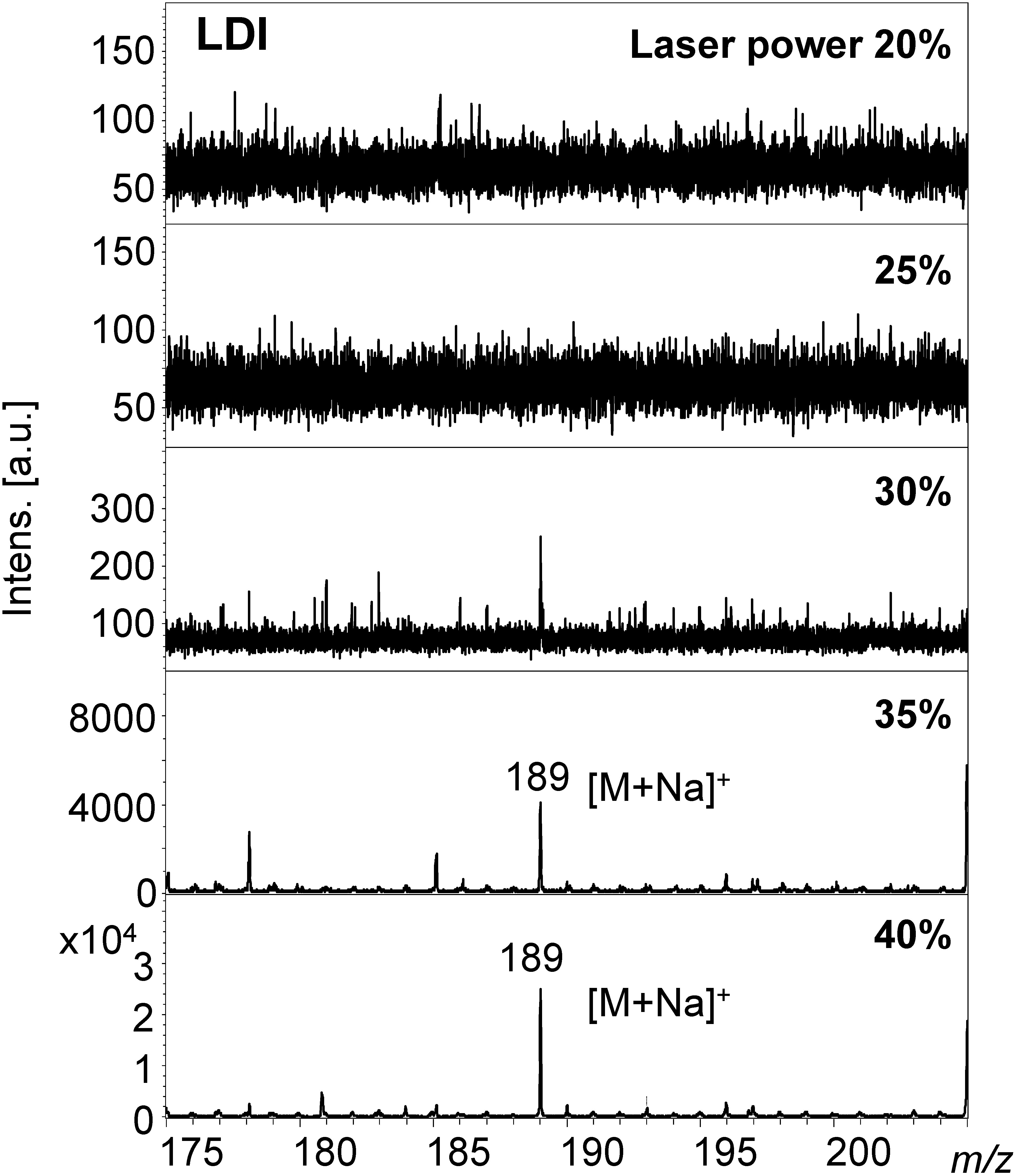
Fig. 5. LDI mass spectra of 3-methylxanthine at 20%–40% laser power.

The sodium adduct ion [M+Na]^+^ at *m*/*z* 189 was observed in the MALDI-MS of 3-methylxanthine, rather than the protonated molecule at *m*/*z* 167 (Supporting Information-4 and 5, and Figs. 4 and 5). A 3-methylxanthine ion was produced even at a low laser power of 20% in DAAQ-MALDI-MS. As the laser power was gradually increased the *S*/*N* ratio of the ion peak also increased (Fig. 4). An ion was not observed at low laser power in LDI-MS, but was produced with a high laser power of 35 or 40% (Fig. 5).

The protonated form of the DAAQ molecule was also observed at *m*/*z* 239 (Fig. 2 and Supporting Information-5). The ion intensity of DAAQ was at a similar level to the signal noise in the DAAQ-MALDI mass spectrum of epinastine at 20% laser power (Fig. 2). In comparison the ion intensity of DAAQ was nearly the same as the analyte in the DAAQ-MALDI mass spectrum of 3-methylxanthine at 40% laser power. Matrix signals generally predominate in MALDI-MS spectra because the matrix compounds strongly absorb laser light. However, DAAQ signals were not predominant in the DAAQ-MALDI-MS of 3-methylxanthine because the absorbance of DAAQ is low at 355 nm which is the wavelength of the laser and because the electron conjugate system is longer than that of conventional matrices.

In the case of camptothencin, a protonated molecule at *m*/*z* 349 was generated at a much lower laser power of 5% in LDI- and DAAQ-MALDI-MS, and there was no effect of DAAQ (Supporting Information-6).

## CONCLUSION

DAAQ effectively assisted in the MALDI ionization of alkaloids as a matrix in both the generation of protonated molecules and sodium adduct ions. Thus, DAAQ improved ionization efficiency of alkaloids in MALDI-MS. DAAQ itself was irradiated by the laser at a wavelength of 355 nm, and DAAQ assisted in the irradiation and desorption of alkaloids. DAAQ did not absorb laser light effectively at 355 nm and its ionization efficiency was not good. This explains why the matrix signals are reduced and the analyte signals from alkaloids are enhanced in MALDI-MS (Fig. 2 and Supporting Information-5). Thus, DAAQ assisted in the ionization of alkaloids in MALDI-MS.

## Notes

Mass Spectrom (Tokyo) 2022; 11(1): A0109
