# Supplementary material for: Diaminoanthraquinone Enhances Alkaloid Ionization in MALDI-MS
Source: Mass Spectrom (Tokyo). 2022 Dec 28;11(1):A0109. doi: 10.5702/massspectrometry.A0109 (PMC9853952; doi:10.5702/massspectrometry.A0109)
Supplement: Supplementary Data [file massspectrometry-11-1-A0109-s001.pdf]

Supporting Information

Diaminoanthraquinone enhances the alkaloid ionization in MALDI-MS.

Tohru Yamagaki* and Tsukiho Osawa
Bioorganic Research Institute, Suntory Foundation for Life Sciences, 8-1-1 Seikadai, Seika-cho, Soraku-gun, Kyoto 619-0284, Japan

SI-1. The UV spectrum of DAAQ.

SI-2. UV-spectra of 100μM DAAQ only and
100μM DAAQ + 40μM Epinastine solution.

SI-3. MALDI Mass Spectra of 100nM Epinastine and 250μM DAAQ.

SI-4. LDI and DAAQ-MALDI mass spectra of
3-methylxanthine at 40% laser power.

SI-5. LDI and DAAQ-MALDI mass spectra of
3-methylxanthine at 40% laser power.

SI-6. LDI and DAAQ-MALDI mass spectra of camptothecin at 5% laser power.

Supporting Information-1.

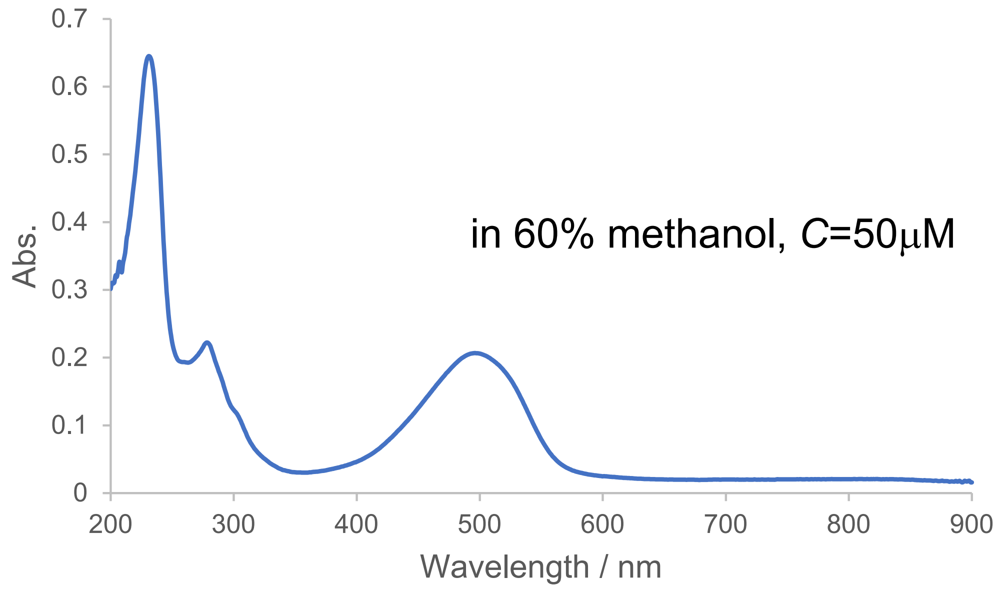

SI-1. The UV spectrum of DAAQ.

Supporting Information-2.

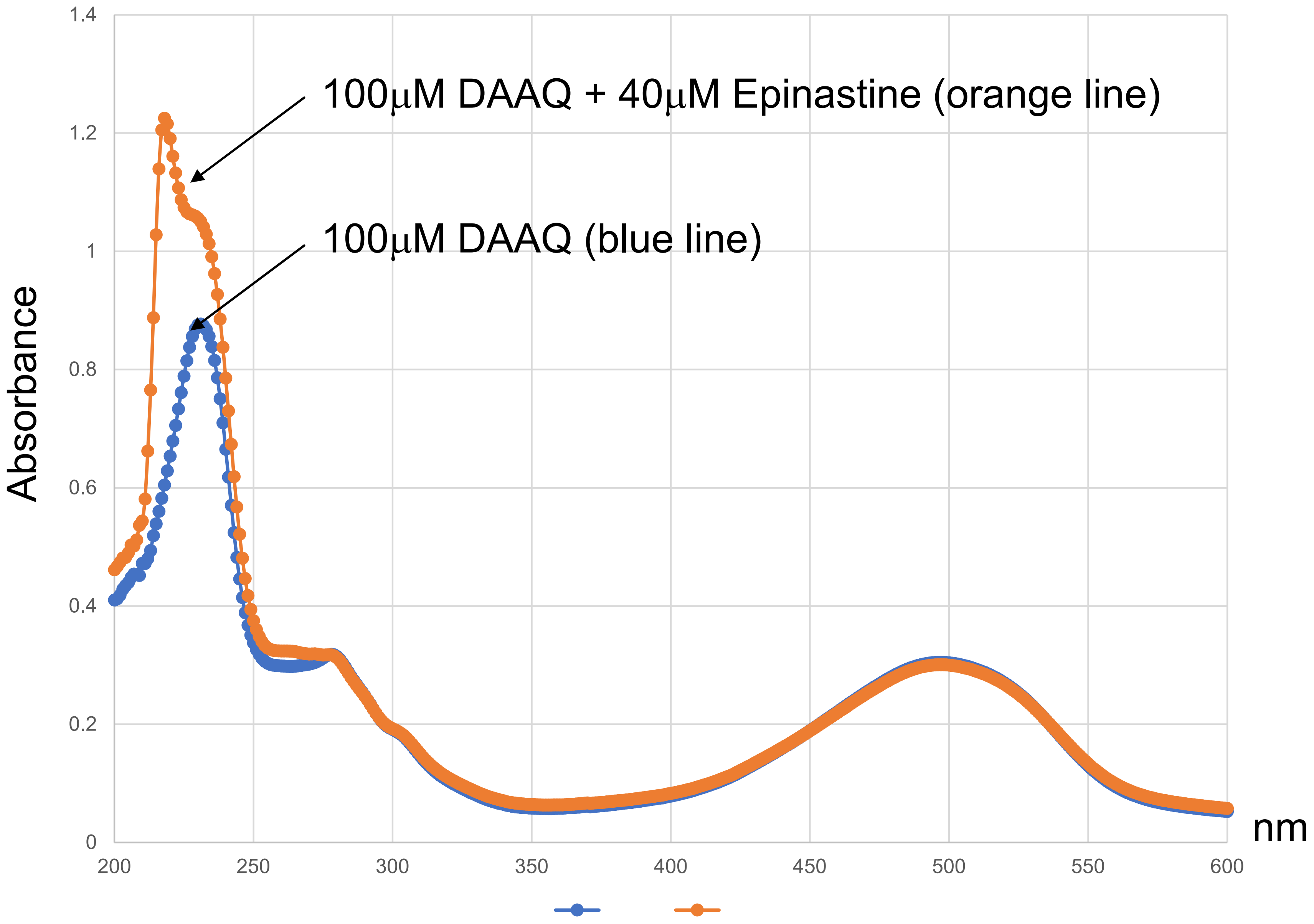

SI-2. UV-spectra of 100μM DAAQ only and 100μM DAAQ + 40μM Epinastine solution.

Supporting Information-3.

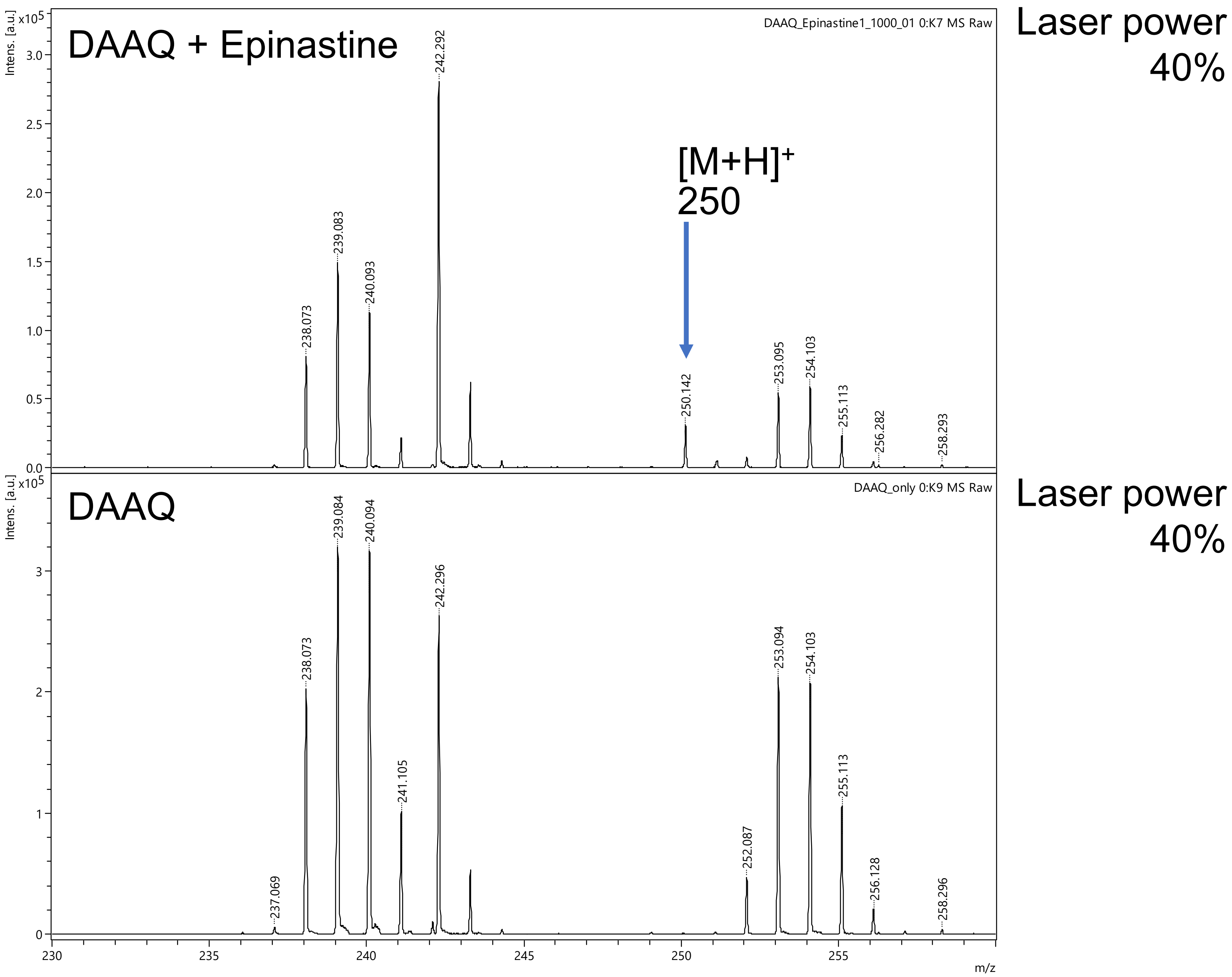

SI-3. MALDI Mass Spectra of 100nM Epinastine and 250μM DAAQ.

Supporting Information-4.

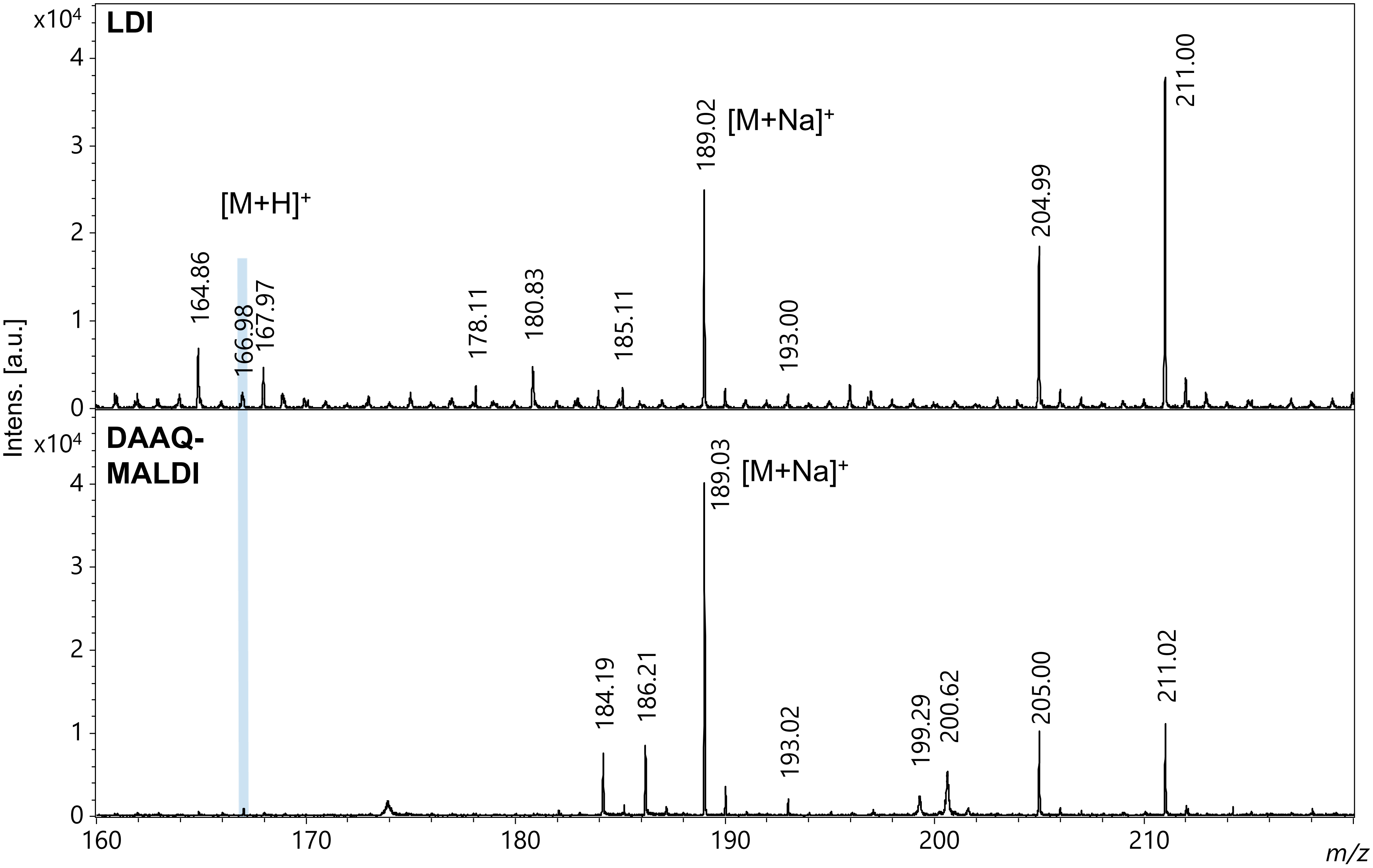

SI-4. LDI and DAAQ-MALDI mass spectra of 3-methylxanthine at 40% laser power.

Supporting Information-5.

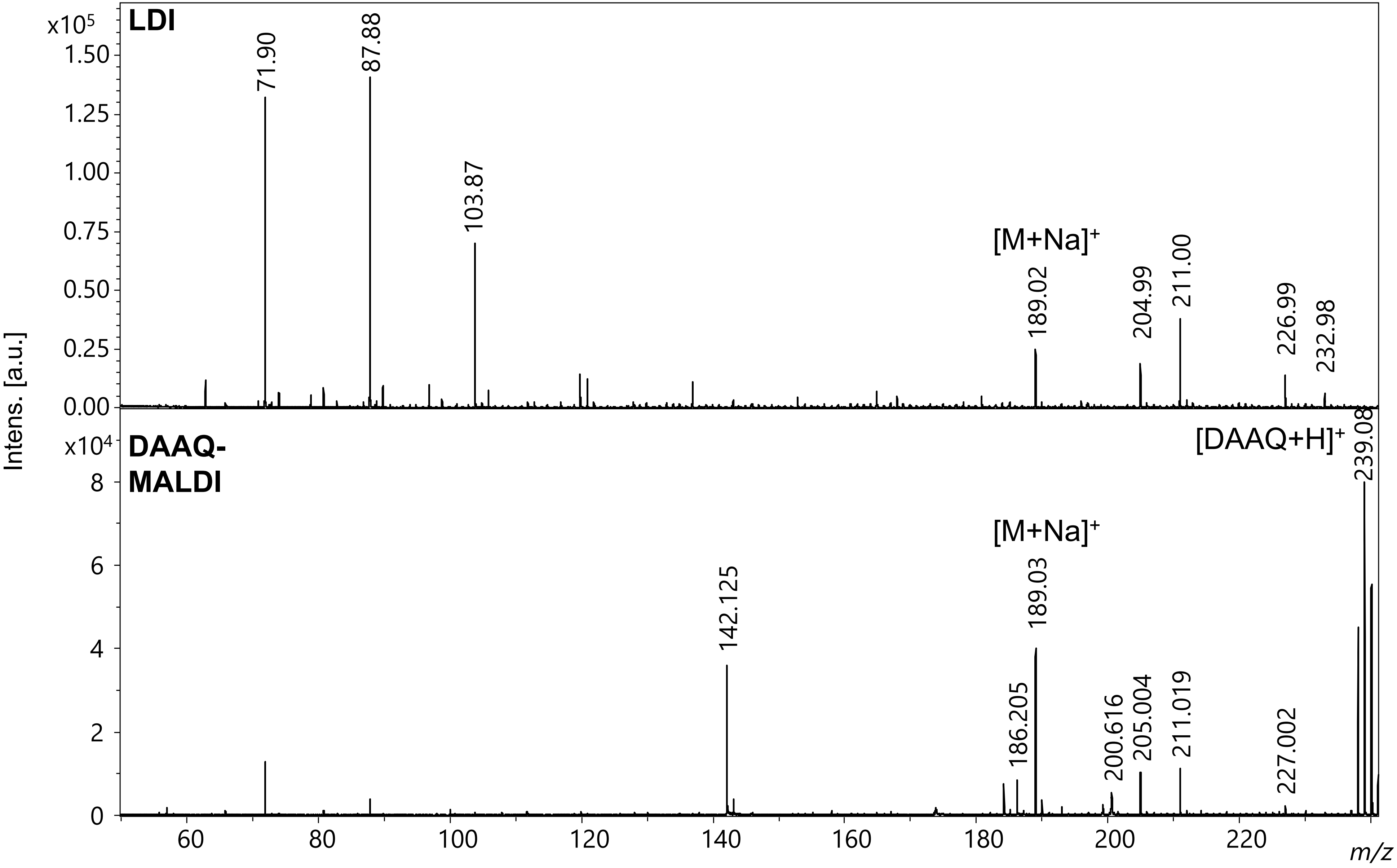

SI-5. LDI and DAAQ-MALDI mass spectra of 3-methylxanthine at 40% laser power.

Supporting Information-6.

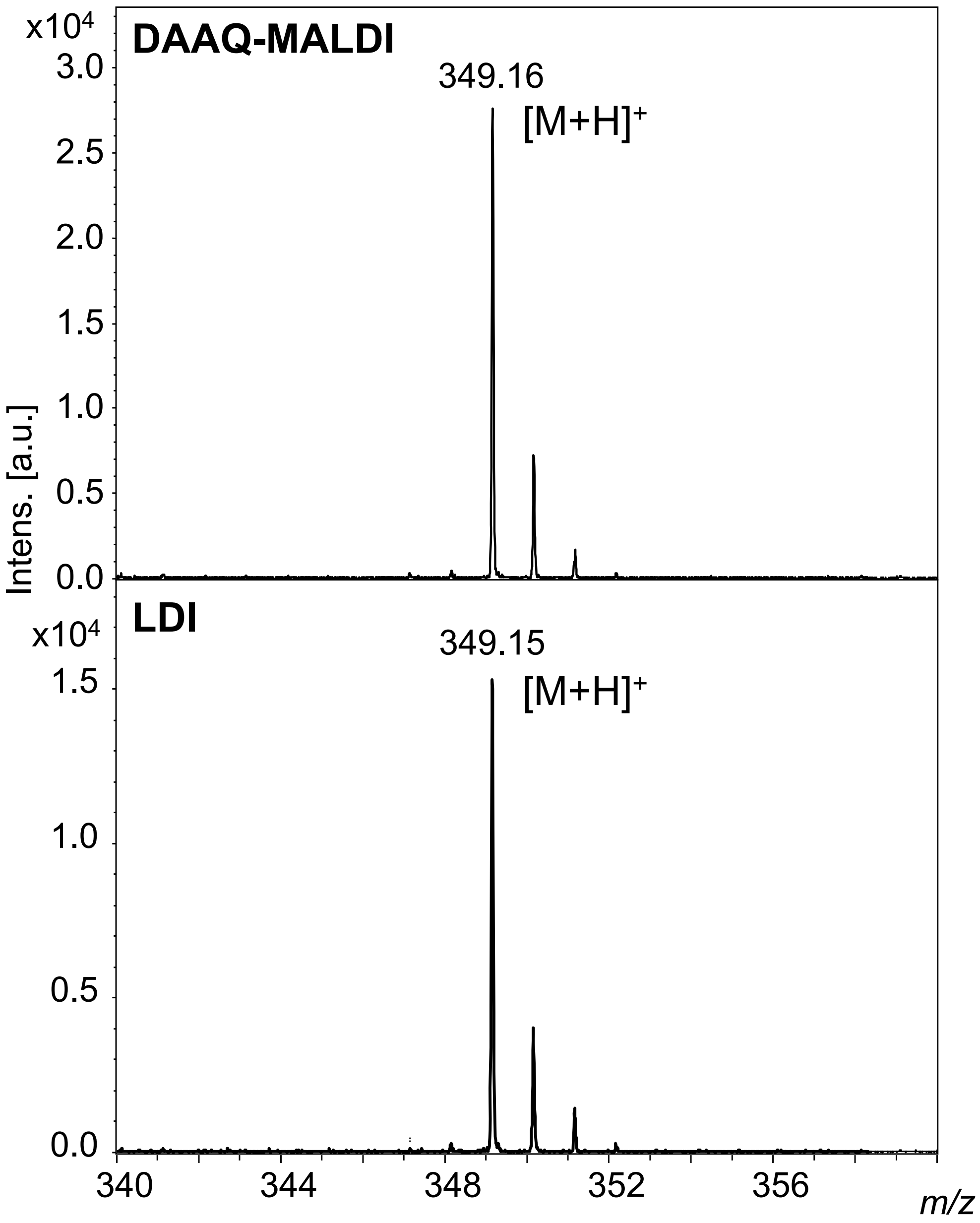

SI-6. LDI and DAAQ-MALDI mass spectra of camptothecin at 5% laser power.
